# Transferosomes Containing 20-Hydroxyecdysone for Psoriasis Treatment: Preparation, Characterization, and In Vitro and In Vivo Toxicity Assessment

**DOI:** 10.3390/ph19081157

**Published:** 2026-07-25

**Authors:** Pawel Bakun, Dariusz T. Mlynarczyk, Kacper Durowicz, Szymon Tomczak, Jolanta Dlugaszewska, Daniel Ziental, Robert Kleszcz, Aleksandra Majchrzak-Celińska, Ewelina Musielak, Mateusz de Mezer, Mikołaj Baranowski, Aneta Wozniak-Braszak, Emilia Cicha, Violetta Krajka-Kuzniak, Anna Jelinska, Tomasz Goslinski, Ludwika Piwowarczyk

**Affiliations:** 1 Department of Chemical Technology of Drugs, Poznan University of Medical Sciences, Rokietnicka 3, 60-806 Poznan, Poland; 87261@student.ump.edu.pl (K.D.); tomasz.goslinski@ump.edu.pl (T.G.); 2 Department of Pharmaceutical Chemistry, Poznan University of Medical Sciences, Rokietnicka 3, 60-806 Poznan, Poland; szymon.tomczak@ump.edu.pl (S.T.); ajelinsk@ump.edu.pl (A.J.); lpiwowarczyk@ump.edu.pl (L.P.); 3 Department of Genetics and Pharmaceutical Microbiology, Poznan University of Medical Sciences, Rokietnicka 3, 60-806 Poznan, Poland; jdlugasz@ump.edu.pl; 4 Department of Inorganic and Analytical Chemistry, Poznan University of Medical Sciences, Rokietnicka 3, 60-806 Poznan, Poland; dziental@ump.edu.pl; 5 Department of Pharmaceutical Biochemistry, Poznan University of Medical Sciences, Rokietnicka 3, 60-806 Poznan, Poland; kleszcz@ump.edu.pl (R.K.); majchrzakcelinska@ump.edu.pl (A.M.-C.); emusielak@ump.edu.pl (E.M.); vkrajka@ump.edu.pl (V.K.-K.); 6 Department of Immunobiology, Poznan University of Medical Sciences, Rokietnicka 8, 60-806 Poznan, Poland; mdemezer@ump.edu.pl; 7 Department of Functional Materials Physics, Institute of Physics, Faculty of Physics and Astronomy, Adam Mickiewicz University, Uniwersytetu Poznańskiego 2, 61-614 Poznan, Poland; mikbar@amu.edu.pl (M.B.); aneta.wozniak-braszak@amu.edu.pl (A.W.-B.); 8 Animal Facility, Poznan University of Medical Sciences, Rokietnicka 8, 60-806 Poznan, Poland; emiliacicha@ump.edu.pl

**Keywords:** hydroxyecdysone, liposomes, nanoparticles, toxicity, zebrafish embryo model

## Abstract

**Background/Objectives**: Psoriasis is a chronic, immune-mediated inflammatory skin disorder affecting millions of individuals worldwide. It remains a therapeutic challenge due to the limited skin penetration of many drugs, adverse systemic effects, and the need for long-term management. Transferosomal nanoformulations containing natural products were proposed as a potential therapeutic tool. **Methods**: Transferosomes containing 20-hydroxyecdysone and resveratrol were prepared using the thin-film hydration method followed by probe ultrasonication, generating several formulation variants differing in composition. The vesicles were characterized by dynamic light scattering (DLS) and nanoparticle tracking analysis (NTA) to determine size, polydispersity index (PDI), and zeta potential. Furthermore, time-domain nuclear magnetic resonance (TD-NMR) relaxation measurements were employed to evaluate local molecular dynamics and membrane fluidity, providing deeper insights into the structural integrity and elasticity of the transferosomal systems. The stability of the nanoformulations was assessed for one month in water and phosphate-buffered saline (PBS). Viability was evaluated in vitro using the MTS assay on human epidermal keratinocyte (HEK) and psoriasis-patient derived human epidermal keratinocyte (PHEK) cell lines, alongside antimicrobial profiling against four representative human skin microbiome strains. Acute toxicity was further examined in vivo using the *Danio rerio* FET test. **Results**: The formulations demonstrated high physicochemical stability over the tested period, maintained desirable particle sizes within 100–200 nm, and showed no cytotoxicity toward skin-associated bacteria or in the zebrafish model. **Conclusions**: The developed nanoformulations present suitable properties in terms of safety and stability and can be considered for potential use in psoriasis treatment.

## 1. Introduction

Psoriasis is a chronic, immune-mediated inflammatory skin disorder affecting millions of individuals worldwide. It is characterized by excessive keratinocyte proliferation, impaired differentiation, and persistent inflammation, leading to erythematous, scaly plaques that significantly reduce patients’ quality of life. The need for long-term management means that, despite the availability of topical agents, systemic therapies, and biologics, due to the limited skin penetration of many drugs and adverse systemic effects, psoriasis remains a therapeutic challenge. These limitations highlight the need for safer and more effective topical delivery systems capable of enhancing dermal drug deposition while minimizing systemic exposure [1,2].

In recent years, ultradeformable vesicular systems, particularly transferosomes, have emerged as promising carriers for transdermal and dermal drug delivery. Transferosomes are flexible phospholipid vesicles containing edge activators that impart high deformability, enabling them to traverse the narrow pores of the stratum corneum and deliver therapeutic agents into deeper skin layers. Their ability to encapsulate both hydrophilic and lipophilic molecules, combined with improved penetration and sustained release, makes them attractive candidates for treating dermatological conditions such as psoriasis. Multiple studies have demonstrated that transferosomes enhance drug bioavailability and improve therapeutic outcomes compared with conventional formulations [3,4].

**Figure 1:**
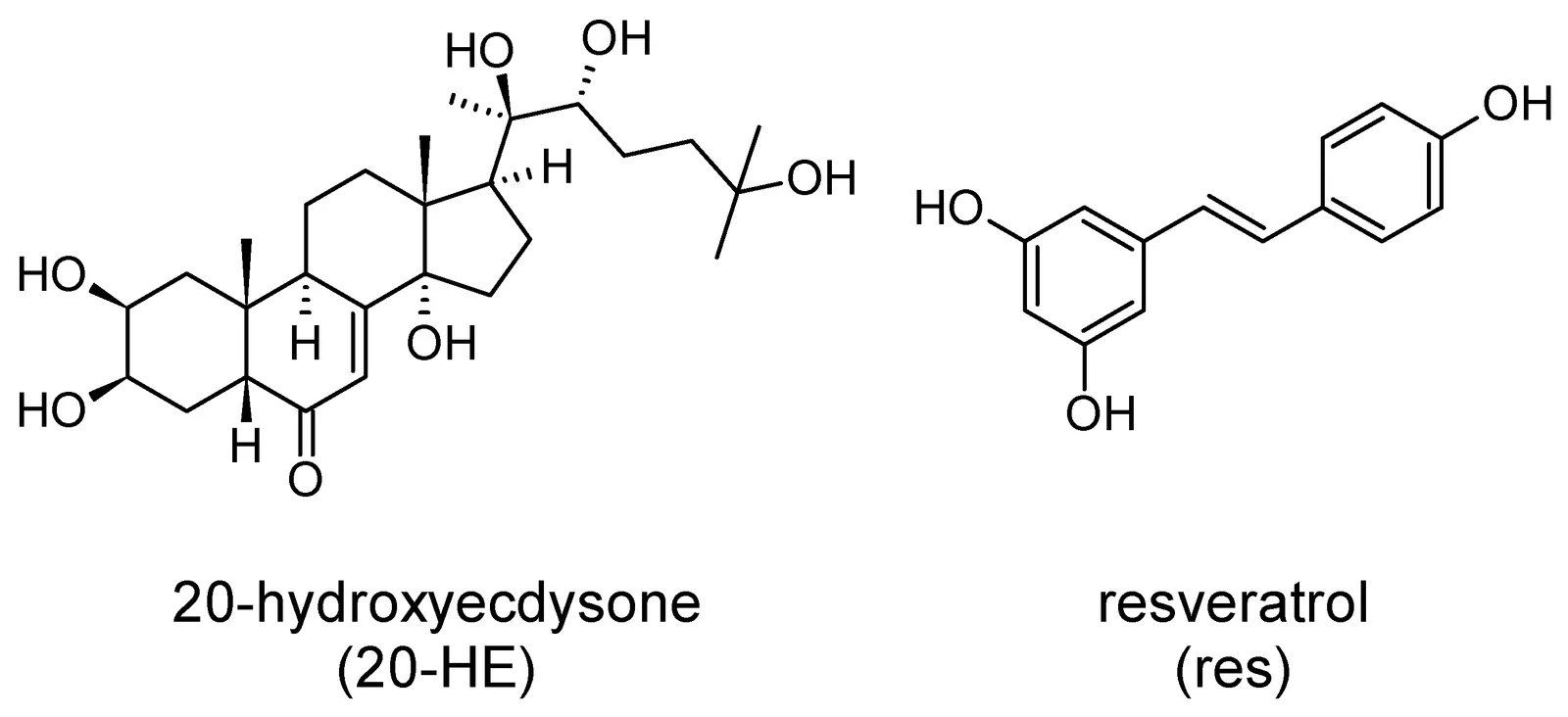
The chemical structure of 20-hydroxyecdysone and resveratrol, used in this study.

Natural bioactive compounds have gained increasing attention as potential anti-inflammatory and regenerative agents in dermatology. Among them, 20-hydroxyecdysone (20-HE; Figure 1)—a phytoecdysteroid known for its cytoprotective, antioxidant, and tissue repair properties—has shown promise for managing inflammatory skin disorders [5]. Experimental studies indicate that 20-HE modulates several molecular pathways involved in chronic inflammation, including the suppression of pro-inflammatory mediators and promotion of tissue homeostasis, while demonstrating a favorable safety profile in mammalian systems [5]. A recent study evaluating a topical nanoformulation of 20-HE in human epidermal keratinocytes (HEKs) and psoriasis patient-derived epidermal keratinocytes (PHEKs) demonstrated good cellular tolerability of the free compound over a broad concentration range, supporting its potential suitability for local dermatological applications [6]. Importantly, preclinical data obtained in an imiquimod-induced psoriasis-like mouse model revealed that 20-HE significantly alleviated psoriatic skin lesions, reduced PASI scores, epidermal hyperplasia, and immune cell infiltration [7]. Moreover, treatment with 20-HE decreased the expression of key cytokines involved in psoriasis pathogenesis, including IL-17A, IL-23, and IL-6, suggesting direct modulation of the IL-23/IL-17 inflammatory axis [5,7]. Given that aberrant keratinocyte activity and dysregulated IL-23/IL-17 signalling are central drivers of psoriasis development and persistence [8,9], the currently available evidence provides a strong rationale for further investigation of topical 20-hydroxyecdysone formulations as a novel therapeutic approach for psoriasis management [7]. However, its clinical application is limited by poor skin permeability and low physicochemical stability, necessitating the development of advanced delivery systems to enhance its therapeutic potential. Recent preliminary studies indicate that transferosome-based formulations can effectively encapsulate 20-HE, improve its stability, and facilitate dermal penetration, supporting their potential use in psoriasis therapy [10].

Resveratrol, a naturally occurring polyphenolic compound found in grapes and the plant *Polygonum cuspidatum*, has also been explored as a supportive agent in dermatological applications. Its well-documented antioxidant and anti-inflammatory properties allow it to modulate key pathways involved in skin inflammation and oxidative stress, both of which are key components of psoriasis. The inhibition of NF-κB signaling, activation of SIRT1 and AMPK pathways, and the suppression of oxidative stress can lead to attenuation of inflammatory responses and normalization of keratinocyte function [2,11]. Experimental studies in cellular and animal models of psoriasis have demonstrated that resveratrol reduces keratinocyte hyperproliferation and decreases the production of pro-inflammatory cytokines involved in the IL-23/IL-17 axis, a central pathway driving psoriatic inflammation [2]. Furthermore, its antioxidant activity contributes to the reduction in reactive oxygen species and oxidative damage, which are increasingly recognized as important contributors to psoriasis development and persistence [2,11]. Recent advances in topical delivery systems, including lipid nanoparticles, hydrogels, and nanocarrier-based formulations, have further enhanced the therapeutic potential of resveratrol by improving its stability, skin penetration, and local bioavailability [11,12]. Despite the promising biological activity of resveratrol, including affecting keratinocyte dysregulation, oxidative stress, and IL-23/IL-17-mediated inflammation in psoriasis pathogenesis [13], its practical use is limited by poor solubility, rapid degradation, and insufficient skin penetration. Taking the above into consideration, resveratrol is a suitable candidate for lipid-based nanocarrier enhancement, which can improve its stability and facilitate more efficient dermal delivery [2,4].

Given the growing interest in combining natural bioactives with nanocarrier-based delivery systems, the present study focuses on the preparation and characterization of 20-hydroxyecdysone-loaded transferosomes, evaluating their physicochemical properties, stability, and biological safety. By integrating modern nanotechnology with bioactive compounds, this work aims to contribute to the development of innovative, effective, and well-tolerated topical treatments for psoriasis.

## 2. Results and Discussion

### 2.1. Physicochemical Properties and Stability Evaluation

**Figure 2:**
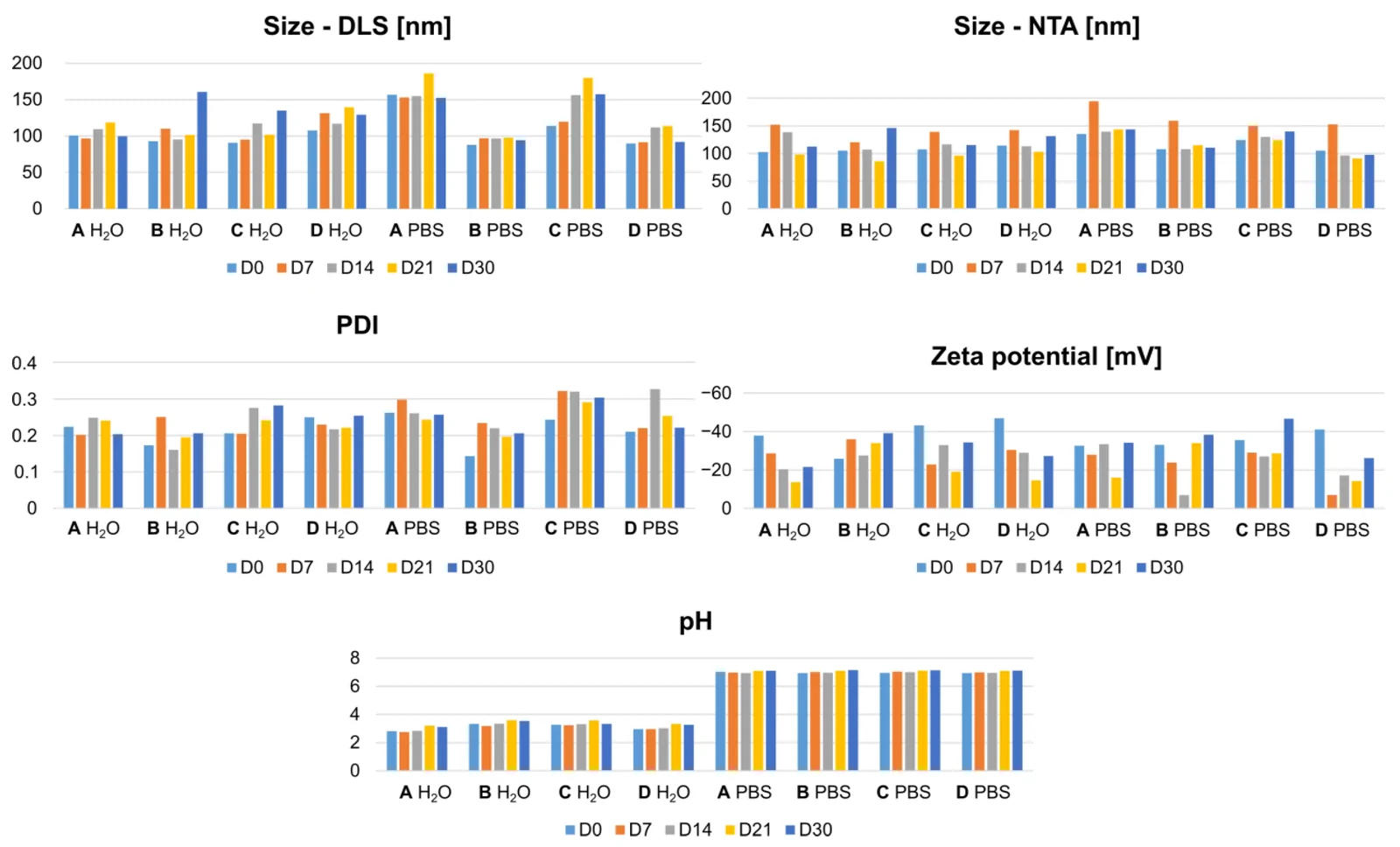
The stability of the tested transferosomal nanoformulations over a one-month period. Dynamic light scattering (DLS, nm), nanoparticle tracking analysis (NTA, nm), polydispersity index (PDI), pH, and zeta potential measurements (mV) of the transferosomal formulations **A**–**D**.

A one-month stability study was conducted using two hydration media, namely deionized water and phosphate-buffered saline (PBS). The nanoformulations were subjected to standard physicochemical characterization, including dynamic light scattering (DLS) to determine the hydrodynamic diameter, polydispersity index (PDI), and zeta potential, as well as nanoparticle tracking analysis (NTA) to assess particle size distribution. Additional parameters such as pH, apparent and actual encapsulation efficiencies were also evaluated to comprehensively characterize the transferosomal systems. The variations in physicochemical parameters, such as particle size (DLS, NTA), the PDI, zeta potential, and pH, over the course of one month are presented in Figure 2 and Table 1.

At the initial time point, the water-hydrated transferosomes exhibited more uniform, narrowly distributed particle sizes than the PBS-hydrated counterparts. This observation is consistent with previous reports indicating that ionic strength and buffer composition can influence vesicle packing, membrane elasticity, and hydration behavior, often leading to broader size distributions in salt-containing media [14].

All formulations demonstrated favorable colloidal characteristics, with polydispersity index (PDI) values below 0.3, indicative of homogeneous nanoscale populations suitable for dermal delivery applications. In most cases, PDI values were around 0.2, which is generally considered optimal for lipid-based nanocarriers and indicative of efficient vesicle formation [15,16].

The zeta potential values ranged from −47 mV to −25 mV for water-hydrated samples and from −41 mV to −32 mV for PBS-hydrated formulations. These values fall within the range typically associated with electrostatically stabilized lipid vesicles, where surface charges below −30 mV are generally sufficient to prevent aggregation through repulsive interactions [17]. The slightly less negative potentials observed in PBS are likely attributable to charge screening by phosphate and sodium ions, a well-documented effect in colloidal systems [18].

**Figure 3:**
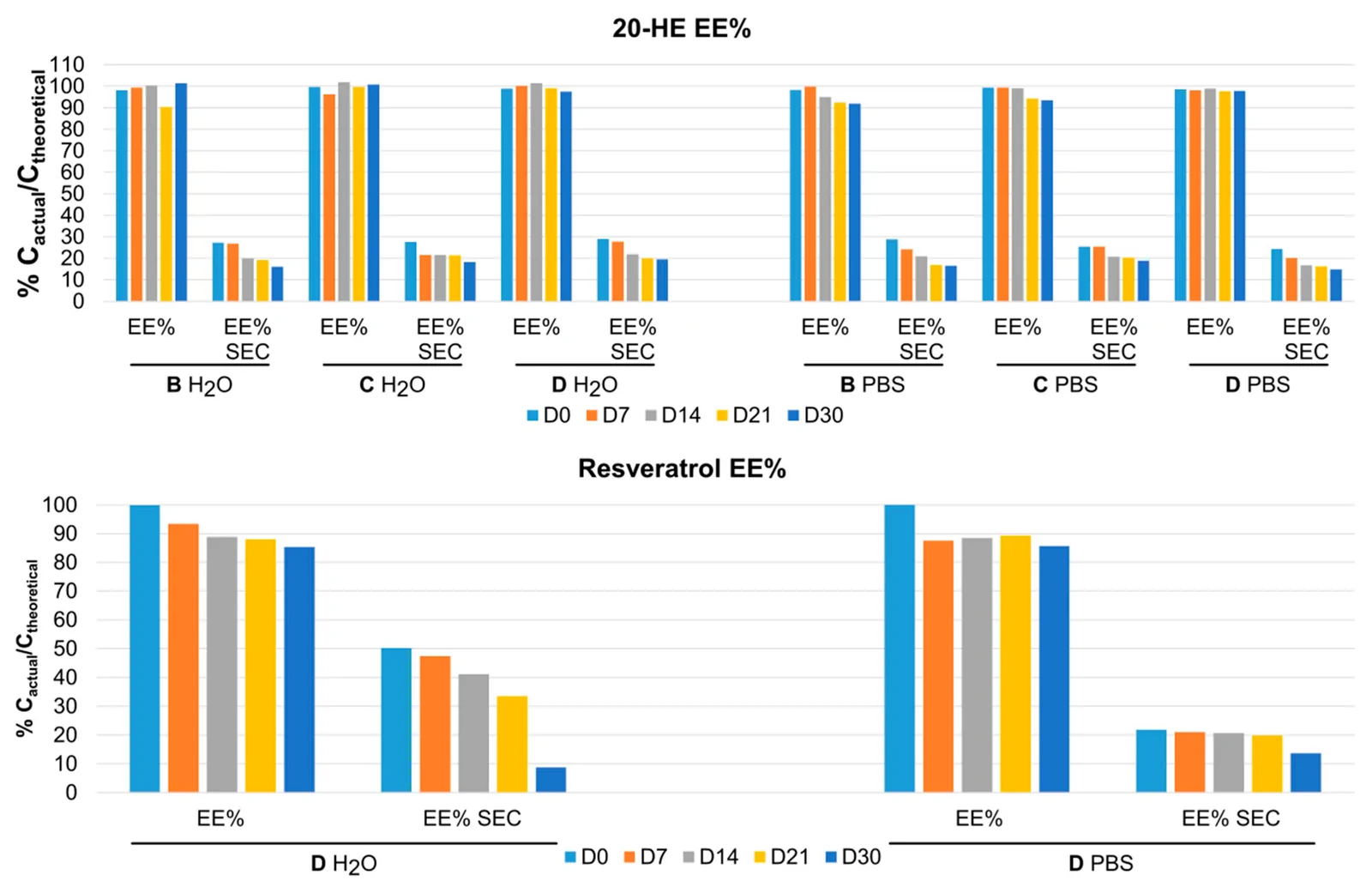
Changes in the content of the active pharmaceutical ingredients (20-HE in formulations **B**–**D** and resveratrol in **D**) over the course of 30-day storage. SEC—size-exclusion chromatography.

The apparent encapsulation efficiency of the active compounds, 20-hydroxyecdysone and resveratrol, was approximately 90% (Figure 3), suggesting a strong initial association of the molecules with the lipid bilayer or aqueous core. However, following size exclusion chromatography (SEC), the actual encapsulation efficiency was determined to be substantially lower, in the range of 25–30%. This discrepancy between apparent and actual EE% values is commonly reported for hydrophilic or amphiphilic compounds, when the non-encapsulated drug may transiently associate with vesicle surfaces [19,20]. The SEC-corrected values therefore more accurately reflect the true loading capacity of the transferosomal systems. This is a crucial aspect in the characterization of the nanoformulation for further research. A scenario in which part of the API is located inside the nanocarriers while another part adheres to their exterior surface should be considered.

Over the course of the thirty-day storage, all of the parameters undergo changes, at times significant.

The pH of the formulations changes only slightly—up to 0.3 for water-hydrated and up to 0.2 for PBS-hydrated formulations. In each case, the pH values increase over time, which might be indicative of the degradation of 20-HE and resveratrol and the formation of less acidic decomposition (oxidation, hydrolysis) products [21,22].

In aqueous medium, most formulations demonstrated moderate size fluctuations over time without a clear monotonic trend. Formulation **A** was relatively stable, showing only minor oscillations; formulation **C** showed a gradual increase, indicating slow aggregation, while formulations **B** and **D** exhibited more pronounced instability, particularly at later time points.

In PBS, significantly different behavior was observed, with clear formulation-dependent effects. Formulation **B** showed remarkable stability across all time points, indicating resistance to ionic destabilization. Formulation **D** remained relatively stable with only a transient increase. In contrast, formulations **A** and **C** exhibited pronounced increases in size and fluctuations.

The particle size data combined with the recorded PDI show that aggregation is not purely linear but often follows a non-monotonic pattern of initial stabilization or minor fluctuations (D0–D7), a progressive increase in size (D7–D21), followed by a partial reduction or plateau (D21–D30). Such behavior indicates that the system undergoes dynamic restructuring rather than simple irreversible aggregation. Possible processes might include reversible vesicle fusion/fission, equilibration between smaller and larger populations, or the redistribution of encapsulated components [23]. The delayed onset of strong aggregation (often after ~14 days, especially in PBS) suggests a “lag phase” of apparent stability, after which structural integrity starts to deteriorate. This is characteristic of lipid-based systems, where slow processes such as lipid oxidation, hydrolysis, or rearrangement can progressively affect membrane properties [24]. The dispersion medium influenced the hydrodynamic diameter of some formulations, particularly **A** and **C**, which exhibited larger particle sizes in PBS than in water. However, PDI values remained comparable throughout the storage period, indicating the preservation of colloidal homogeneity. No consistent medium-dependent effect was observed for zeta potential. Overall, the formulations maintained satisfactory physicochemical stability in both media, as neither of the studied parameters for the corresponding formulations exhibited statistical significance (*p* > 0.05).

The zeta potential measurements provided further insight into the colloidal stability of the investigated nanoformulations. The initial values were predominantly in the range of −30 to −45 mV, indicating relatively stable dispersions at the time of preparation, as systems exceeding ±30 mV are generally considered electrostatically stabilized [25]. However, a clear decrease in the absolute zeta potential values was observed over time for several formulations. In particular, some systems showed a pronounced reduction in zeta potential magnitude to approximately −7 mV, which corresponds to a highly unstable colloidal state due to insufficient electrostatic repulsion. This decrease slightly correlates with the increase in particle size observed in the DLS measurements.

Importantly, the effect was more pronounced in PBS, which can be explained by ionic strength-induced screening of surface charges. The presence of electrolytes compresses the electrical double layer, reducing the effective zeta potential and interparticle repulsion, thereby promoting aggregation [26]. Additionally, the observed fluctuations in zeta potential over time further support the presence of dynamic restructuring processes within the system.

These findings are consistent with the encapsulation efficiency assessment of the formulations. In each of the tested formulations (**B**–**D**, both in water and PBS), the apparent encapsulation of 20-HE remains unchanged or decreases by no more than 10% over the whole study period. In the case of EE% SEC, the changes are more pronounced, with half of the initial EE% SEC values being lost over one month of storage. This shows that 20-HE is still present in the formulation but is gradually being expelled from within the double membrane of the transferosome, most likely due to the structural changes described above. In case of resveratrol in **D**, significant changes occur when water is used as a dispersing medium: the initial higher EE% SEC decreases over time, and the final concentration is lower than that in PBS at the end of the study. This phenomenon might be explained by the chemical structure of resveratrol, which contains acidic phenol groups that might be more ionized in PBS, greatly decreasing its affinity for lipids (lower initial EE% SEC) but at the same time reducing its mobility through the vesicle membrane, thus stabilizing its actual concentration in transferosomes over time.

Another interesting finding is the increased stability of both compounds in the developed formulation, as 20-HE and especially resveratrol are known to be highly prone to degradation in aqueous media, particularly in slightly alkaline PBS conditions [21,27]. As both compounds are poorly soluble in water, there seems to be an interaction between un-encapsulated compounds and the surface of lipid membranes which acts in a protective manner.

### 2.2. In Vitro Cytotoxicity

The cytotoxic activity of nanoformulations (**A**–**D**) was evaluated in HEK and PHEK cell lines using the MTS assay after 24, 48, and 72 h of incubation at concentrations ranging from 5 to 50 µM. In all tested conditions, IC_50_ values exceed 50 µM, indicating relatively low cytotoxicity of the studied nanoformulations. These findings are in line with literature reports, where 20-HE is reported as non-toxic in a concentration range of 5–50 µM [28].

**Figure 4:**
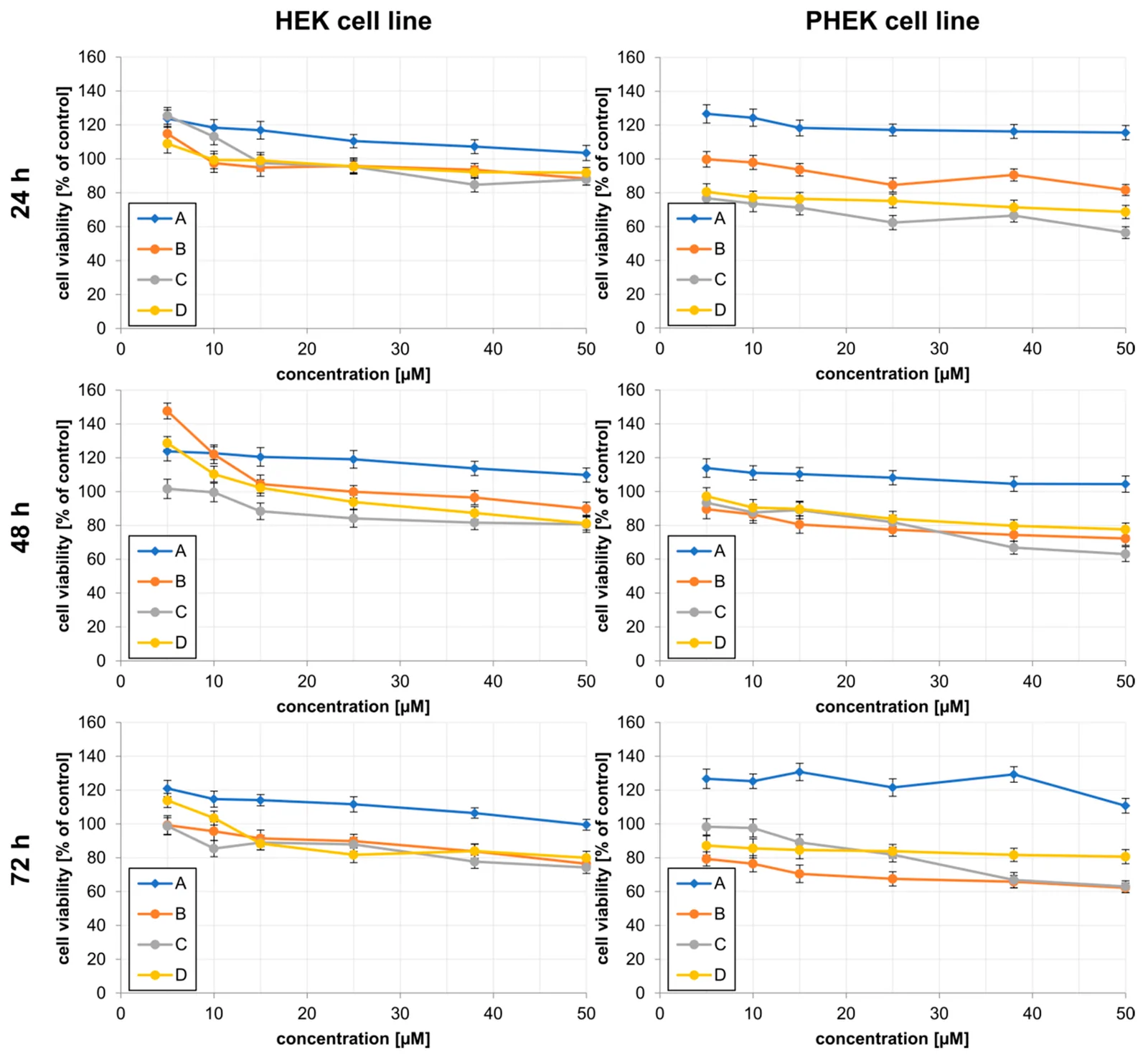
HEK and PHEK cell viability upon exposure to different concentrations of API in formulations **A**–**D** after 24, 48 and 72 h of incubation. Error bars indicate standard error of the mean.

In HEK cells, the tested nanoformulations generally demonstrated very limited or no cytotoxic effects. Formulation **B** maintained cell viability close to or above 90% at most concentrations and time points (Figure 4). Moreover, increased metabolic activity was observed at lower concentrations (5–10 µM), particularly after prolonged incubation, suggesting possible stimulation of cellular metabolic activity. Formulations **C** and **D** showed modest cytotoxic effects, especially at 50 µM after 48 h and 72 h, where cell viability decreased to approximately 74–81%. Nevertheless, none of the tested formulations reduced viability below 70% in both HEK and PHEK cells.

In contrast, PHEK cells exhibited higher sensitivity to the tested nanoformulations. The strongest reduction in viability was observed for formulation **C**, which decreased PHEK viability to approximately 56% after 24 h at 50 µM. A concentration-dependent reduction in viability was also observed for **D**, with viability values ranging from approximately 69 to 81% depending on concentration and incubation time. Formulation **B** exerted a milder effect in PHEK cells, maintaining viability above 80% in most cases.

Overall, the obtained results indicate that the analyzed nanoformulations exhibit low to moderate cytotoxicity, with stronger biological effects observed in PHEK cells compared to HEK cells. Among the tested formulations, **C** and **D** demonstrated the greatest antiproliferative potential.

### 2.3. Antimicrobial Profiling

The antibacterial properties of the tested formulations were evaluated against two strains of *Staphylococcus epidermidis* (standard and clinical strain), dominant bacterial species in the human skin, and two strains of *Staphylococcus aureus* (standard and clinical strain), a prevalent commensal bacterium and part of the normal human skin microbiota in approximately 30% of the human population [29]. The conducted studies did not reveal any antimicrobial effect of the tested formulations against either *Staphylococcus epidermidis* or *Staphylococcus aureus* strains at the applied concentrations. Accordingly, both the minimum inhibitory concentration (MIC) and minimum bactericidal concentration (MBC) values were determined to be above the highest tested concentration, i.e., > 0.5 mM (see Table 2).

From the perspective of developing topical treatments for psoriasis, the lack of antibacterial activity against standard and clinical strains of the skin microbiota is a significant advantage. Psoriasis-affected skin exhibits a severely compromised epidermal barrier and a frequently dysregulated microbiome [30]. Therapeutic formulations that possess aggressive, broad-spectrum antimicrobial properties risk eradicating beneficial commensal bacteria, such as *S. epidermidis*, which can aggravate inflammation and lead to opportunistic pathogen overgrowth. Therefore, the antimicrobial neutrality of the designed transferosomes confirms their biocompatibility with the natural skin microenvironment. This ensures that the vehicle can safely deliver 20-hydroxyecdysone and resveratrol to deeper epidermal layers without disrupting the delicate microbiological homeostasis of the patient’s skin.

### 2.4. In Vivo Toxicity

Exposure of zebrafish embryos to transferosomal nanoformulations of resveratrol, 20-hydroxyecdysone, and the free compounds at concentrations up to 20 µM resulted in no detectable toxicity (Table 3). These findings demonstrate the safety and biocompatibility of both the active compounds and their nanoformulated forms in the zebrafish model, supporting their suitability for further biological and pharmacological investigation.

### 2.5. ^1^H Nuclear Magnetic Resonance Analysis

**Figure 5:**
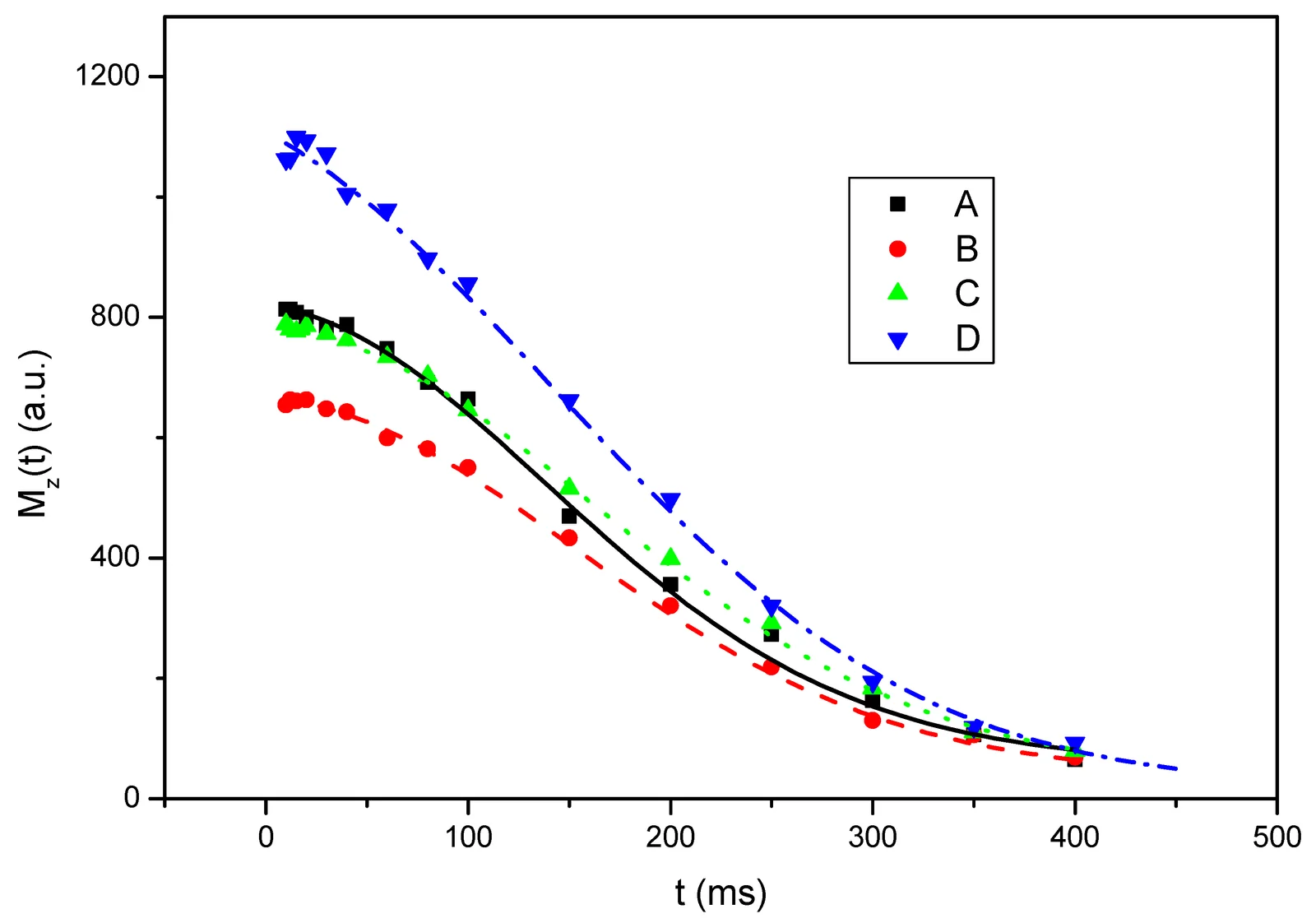
Magnetization decay curves obtained during spin–spin relaxation time (T_2_) measurements for transferosomal formulations **A**, **B**, **C**, and **D**.

Analysis of the obtained NMR relaxation parameters revealed that the investigated transferosomal formulations differed mainly in their local molecular organization and dynamic heterogeneity, while the overall spin–lattice relaxation behaviour remained relatively unchanged. The determined values of the relaxation times T_1_ and T_2_ are presented in Table 4. Magnetization decay curves obtained during spin–spin relaxation time (T_2_) measurements for transferosomal formulations **A**, **B**, **C**, and **D** are shown in Figure 5.

The T_1_ relaxation times were very similar for all investigated systems and ranged from 2.17 to 2.30 s. This indicates that the overall spin–lattice relaxation mechanism and global molecular mobility were not significantly affected by formulation composition. Therefore, the incorporation of 20-hydroxyecdysone, the increased concentration of Tween 20, or the simultaneous presence of 20-hydroxyecdysone and resveratrol did not substantially influence the molecular motions governing T_1_ relaxation.

More pronounced differences were observed for the T_2_ relaxation parameters and the relative contributions of the Gaussian and Lorentzian components. In all formulations, the Gaussian component predominated, suggesting that the transferosomal systems contained a dominant fraction of relatively ordered and motionally restricted proton environments. However, the contribution and relaxation behaviour of the Lorentzian component varied depending on the formulation composition, indicating differences in the amount and mobility of more dynamic molecular domains [1,3,4].

Formulation **A**, used as the reference system, exhibited a 79% contribution of the Gaussian component and a 21% contribution of the Lorentzian component, with T_2G_ = 198 ms and T_2L_ = 434 ms. These values indicate a moderately ordered transferosomal structure containing both rigid and mobile domains with intermediate molecular mobility.

The incorporation of 20-hydroxyecdysone in formulation **B** increased the contribution of the Gaussian component to 85%, while both T_2G_ and T_2L_ slightly increased compared to formulation **A**. The increase in T_2_ relaxation times may suggest somewhat enhanced local molecular mobility and more efficient motional averaging of dipolar interactions within the transferosomal formulation. Simultaneously, the increased contribution of the Gaussian component may indicate partial structural ordering induced by intermolecular interactions involving 20-hydroxyecdysone.

Formulation **C**, containing a higher concentration of Tween 20, exhibited the highest T_2L_ value (765 ms), despite the relatively small contribution of the Lorentzian component (12%). According to literature reports, increased molecular mobility results in more efficient averaging of dipolar interactions, leading to slower transverse relaxation and longer T_2_ relaxation times [31,32]. Therefore, the pronounced increase in T_2L_ suggests the presence of a small but highly mobile fraction within the formulation. This observation is consistent with the known role of Tween 20 as an edge activator that enhances transferosomal deformability and flexibility [1,3,4].

The most distinct relaxation behaviour was observed for formulation **D** containing both 20-hydroxyecdysone and resveratrol. This formulation exhibited the highest contribution of the Lorentzian component (30%), while simultaneously showing a markedly shortened T_2L_ value (199 ms). Although the relative amount of the mobile fraction increased, the shorter T_2L_ indicates that molecular mobility within this fraction was locally restricted. Shortening of T_2_ relaxation times is associated with stronger local dipolar interactions and faster dephasing of transverse magnetization [31,32]. This behaviour may therefore suggest increased intermolecular interactions or enhanced structural heterogeneity within the transferosomal formulation. Due to its hydrophobic and aromatic character, resveratrol may interact with transferosomal components and locally influence the molecular organization and dynamic behaviour of the system [4].

Overall, the obtained results indicate that formulation composition mainly influences local molecular dynamics and the balance between rigid and mobile domains within the transferosomal systems. Longer T_2_ values correspond to higher molecular mobility and more efficient motional averaging of dipolar interactions, whereas shorter T_2_ values indicate restricted molecular motion and stronger local intermolecular interactions. Among the investigated formulations, formulation **C** exhibited the highest mobility within the Lorentzian fraction, while formulation **D** showed the strongest local restrictions of molecular mobility and the most heterogeneous dynamic behaviour.

## 3. Materials and Methods

### 3.1. Materials

All reagents used in this study were obtained from reputable suppliers and were used without further purification. Phospholipids such as Phospholipon 80H were purchased from Lipoid GmbH (Ludwigshafen, Germany), while Tween 20 was supplied by Glentham Life Sciences Ltd. (Corsham, UK). Organic solvents, including chloroform and methanol, were of HPLC grade and obtained from various suppliers, such as POCH (Avantor Performance Materials Poland S.A., Gliwice, Poland), Chempur (Piekary Śląskie, Poland), and Chemsolve^®^ (WITKO Sp. z o.o., Łódź, Poland). Sterile water for irrigation used for the hydration of formulations and further dilutions was purchased from B. Braun Medical AG (Sempach, Switzerland), meeting pharmacopeial purity requirements. The active compounds 20-hydroxyecdysone (CAS 5289-74-7; 20-HE) and resveratrol (CAS 501-36-0; res) were supplied from Carl Roth GmbH (Karlsruhe, Germany) and Fluorochem (Hadfield, UK), respectively.

### 3.2. Transferosome Preparation

Transferosomes were prepared using the thin-film hydration technique followed by probe sonication. Briefly, phospholipids (Phospholipon 80H), selected edge activator (Tween 20) and active compounds (20-hydroxyecdysone and/or resveratrol) were dissolved in an organic solvent (Phospholipon 80H stock solution concentration 25 mg/mL in chloroform, Tween 20 stock solution concentration 25 mg/mL in methanol, 20-HE stock solution concentration 10 mg/mL in methanol, resveratrol stock solution concentration 10 mg/mL in methanol) and evaporated under reduced pressure to form a thin lipid film. Tween 20 was used as an edge activator at concentrations of 1–3 mM, corresponding to approximately 5–15 mol% relative to phospholipid (20 mM). This range was selected based on literature reports and preliminary optimization studies, as it provided homogeneous nanosized vesicles with low PDI values, high encapsulation efficiency, and satisfactory colloidal stability while preserving vesicle integrity [33,34]. Organic solvents were removed using a rotary evaporator (Heidolph Instruments GmbH & Co. KG, Schwabach, Germany, equipped with an automatic lift and a diaphragm vacuum pump) at a water bath temperature of 40 °C. The pressure was initially reduced to 250 mbar and maintained until complete evaporation of chloroform (about 30 min). Subsequently, the pressure was further decreased to 70 mbar to remove methanol (about 30 min). Finally, the obtained lipid film was additionally dried under maximum vacuum (approximately 2–6 mbar) for 10 min to ensure complete removal of residual solvents. The dry film was subsequently hydrated with an aqueous phase (water for injection—Braun or PBS buffer) and after 1 h of orbital mixing in 40 °C subjected to probe sonication (Bandelin SONOPULS ultrasonic homogenizer, Berlin, Germany) to obtain homogenous nanosized vesicles. Based on the obtained results, the shortest time required to produce a uniform dispersion was determined to be 5 min in pulse mode (5 s on/5 s off) at a 30% probe amplitude. The detailed composition of each transferosomal formulation variant is shown in Table 5. Physicochemical characterization included measurements of particle size (nm), the polydispersity index (PDI), zeta potential (mV), pH, and the encapsulation efficiency (EE%) of the active compound that was quantified using high-performance liquid chromatography.

### 3.3. Physicochemical Characterization of the Nanoparticles

Particle size and the polydispersity index (PDI) were measured using dynamic light scattering (Malvern Panalytical Zetasizer Ultra, Malvern, UK). Zeta potential was recorded on the Malvern Panalytical Zetasizer Ultra using samples diluted in the same way as for the DLS measurements in folded capillary zeta cells. In addition, particle size distribution was further analyzed using nanoparticle tracking analysis (NTA) with a NanoSight LM10 instrument (Malvern Panalytical, Malvern, UK) to complement DLS measurements and provide single-particle tracking data. The pH of the original, undiluted transferosomal nanoformulations was measured using a Mettler Toledo SevenCompact pH meter (Schwerzenbach, Switzerland).

**Figure 6:**
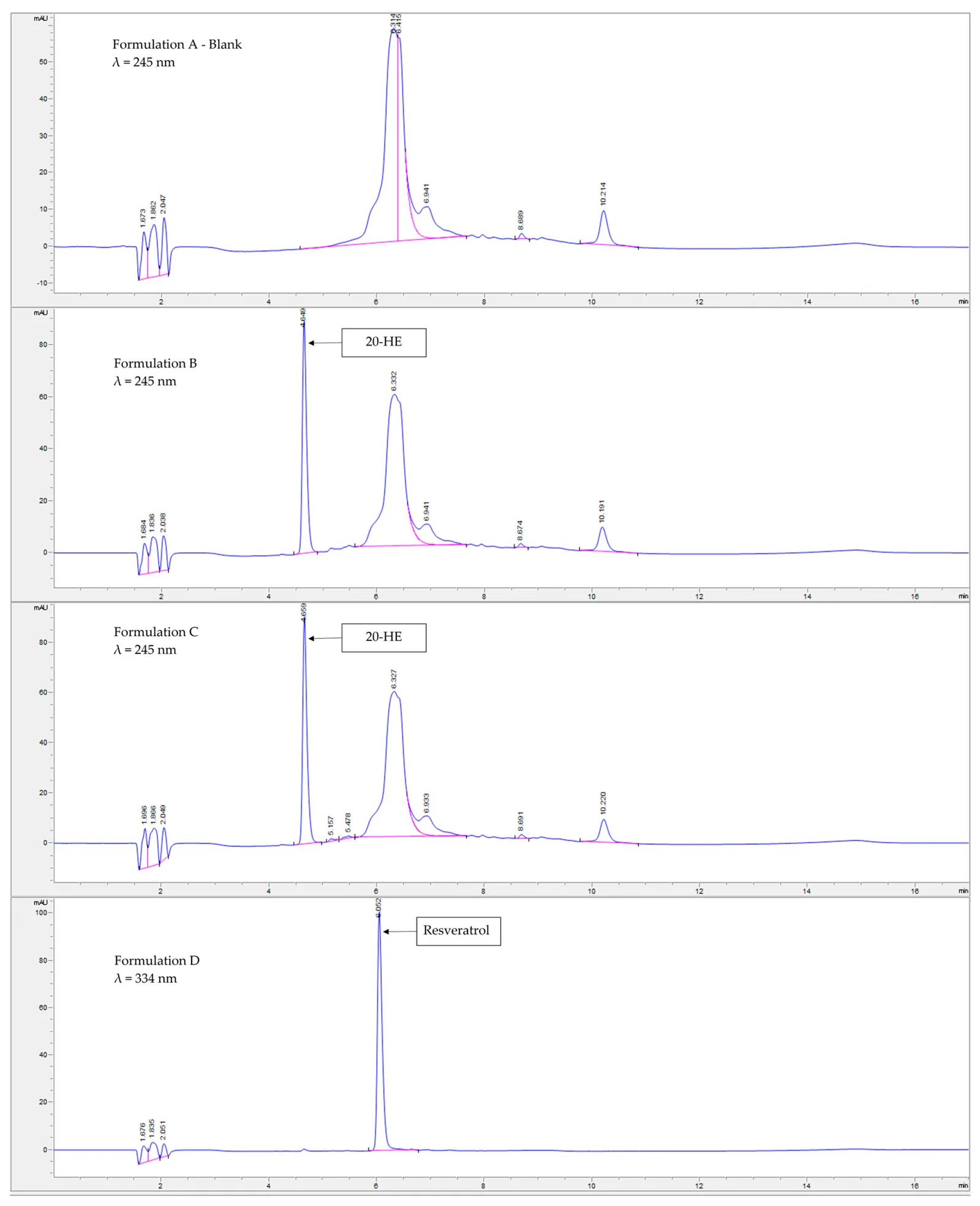
Representative HPLC chromatograms of formulations A–D used for the determination of the encapsulation efficiency of 20-hydroxyecdysone and resveratrol.

The encapsulation efficiency (EE) of the active substance within the nanoformulation was quantified using high-performance liquid chromatography coupled with UV detection. The encapsulation efficiency (EE%) was calculated as the ratio of the amount of compound encapsulated within the nanocarrier to the initial amount of compound used in the formulation, multiplied by 100% [35]. Analyses of content and stability were performed on an Agilent 1260 Infinity II liquid chromatography system (Agilent Technologies, Böblingen, Germany), equipped with a quaternary solvent delivery pump with an integrated degasser, an autosampler, and a diode array detector (DAD). Validation of the HPLC method concerned selectivity, precision, linearity, range, and limits of detection and quantitation [36]. A summary of validation parameters is presented in Table 6, and the representative chromatograms are presented in Figure 6.

To quantify the fraction of the drug incorporated into the nanocarrier, the formulation was purified using commercially available disposable size exclusion chromatography (SEC) columns (PD MiniTrap™ G-25, Cytiva, UK). In accordance with the manufacturer’s protocol, the columns were conditioned prior to use, and purification was achieved through centrifugation, yielding a formulation devoid of free, non-encapsulated compounds. At the same time, the content of active compounds was determined without sample purification (the sum of enclosed and free active substances), called apparent encapsulation.

Because of the complex formulation matrix and the inability to inject the sample directly into the chromatographic system, 100 µL of the nanoformulation was firstly dissolved in 900 µL of HPLC-grade methanol. This solvent was selected to ensure complete disruption of the nanostructure while simultaneously dissolving both the active ingredient and excipients. The resulting solution was analyzed on a reversed-phase C18 HPLC column LiChrospher^®^ RP-18 (5 µm) (Merck, Darmstadt, Germany) under gradient elution conditions.

The mobile phase consisted of solvent A, an aqueous solution containing 0.1% (*v*/*v*) phosphoric acid, and solvent B, chromatographic-grade acetonitrile (ACN). The gradient program began with a composition of 88:12 (A:B), which was linearly adjusted to 20:80 over 7 min at a flow rate of 0.75 mL/min. Subsequently, the system was returned to the initial mobile phase composition over 5 min and maintained under isocratic conditions for an additional 5 min. The overall analysis time was 17 min.

20-HE showed pronounced UV absorbance, with a maximum at approximately 245 nm, while resveratrol was analyzed at 334 nm. All solvents used for chromatographic analysis were of analytical grade and obtained from Avantor Performance Materials Poland S.A. (Gliwice, Poland). The obtained results were expressed as percentages (%) relative to the theoretical initial concentration of the compound in the nanocarrier, i.e., 1 mM (0.48 mg/mL and 0.228 mg/mL for 20-HE and resveratrol, respectively).

Formulations **A**, **B**, **C**, and **D**, each prepared in two variants (hydrated with either water or PBS), were subjected to a series of stability assessments over a one-month period. Measurements were performed at the following time points: D0 (day of preparation), D7 (1 week), D14 (2 weeks), D21 (3 weeks), and D30 (1 month). The stability evaluation included dynamic light scattering (DLS) analysis of particle size, polydispersity index (PDI), and zeta potential, nanoparticle tracking analysis (NTA) for particle size distribution, pH measurements, and the determination of encapsulation efficiency (EE%) using both apparent encapsulation and size exclusion chromatography (SEC).

### 3.4. In Vitro Viability MTS Assay

Human epidermal keratinocyte (HEK) and psoriasis patient-derived keratinocyte (PHEK) cells were maintained in KGM-Gold™ Keratinocyte Growth Medium BulletKit™ (Lonza, Walkersville, MD, USA) supplemented with 10% fetal bovine serum (FBS).

Cells were cultured under standard conditions (37 °C, 5% CO_2_, 95% humidity). The CellTiter 96^®^ AQueous One Solution Cell Proliferation Assay (MTS; Promega, Madison, WI, USA) was used according to the manufacturer’s recommendations to assess the effects of the studied nanoformulations on cell viability. Briefly, the cells were seeded in 96-well plates (5000 cells/well). After 24 h, the growth medium was replaced with fresh medium containing nanoformulations **A**–**D** at API (**20-HE**, **res**) at concentrations ranging from 5 to 50 µM, and the cells were incubated for an additional 24, 48, and 72 h. Then, cells were washed with PBS, and 120 µL of freshly prepared MTS solution in complete medium (20 µL of MTS solution + 100 µL of medium) was directly added to each well. After 1 h of incubation, the absorbance signal (490 nm) was measured using Tecan SPARK (Multi-mode Microplate Reader, Tecan Trading AG, Mannedorf, Switzerland). The assay was performed in triplicate, with three replicates per assay.

### 3.5. Antimicrobial Activity Assessment—Skin Microbiome

The Minimal Inhibitory Concentration (MIC) and Minimal Bactericidal Concentration (MBC) of the tested formulations were determined against representatives of the bacterial community of the skin microbiota: standard strains of *Staphylococcus epidermidis* ATCC 49134 (*S. epidermidis*) and *Staphylococcus aureus* ATCC 6538 (*S. aureus*), and clinical strains of *Staphylococcus epidermidis* and *Staphylococcus aureus*. The microdilution method was used in the performed studies. Bacterial cultures were grown on Tryptic Soy Agar (TSA; OXOID, UK) at 35 ± 1 °C for 18 h. Several colonies were selected and suspended in Mueller–Hinton broth (MHB; Oxoid, UK), and the suspension turbidity was adjusted to a 0.5 McFarland standard. Next, the suspensions were diluted in MHB to obtain a final inoculum containing about 1 × 10^6^ CFU/mL. The formulations were serially diluted (2-fold) in the MHB in 96-well plates, and the microbial suspensions were added. The compounds were tested in the final concentration range of 0.5 mM to 0.007 mM. This concentration range was determined by the experimental setup. The transferosome suspension contained the active compound at a concentration of 1 mM, while the culture medium had to constitute at least 50% of the final assay volume to maintain bacterial viability. Consequently, the highest achievable concentration of the tested compounds was 0.5 mM. Lower concentrations were obtained through successive two-fold serial dilutions. The final microbial inoculum was approximately 5 × 10^5^ CFU/mL. The tests were incubated at 35 ± 1 °C for 18 h. Media without the strains added to the different concentrations of the tested formulations and media inoculated with microbial suspension were used as a negative control and growth control, respectively. All tests were performed in duplicate. The MIC was defined as the lowest concentration at which visible growth was inhibited.

The MBC was determined as an extension of the MIC test. After performing the MIC test and recording the MIC endpoint, every well that demonstrated no growth (concentration equal to or greater than MIC) was subcultured on TSA. The plates were incubated at 35 ± 1 °C for 18–24 h. The MBC was defined as the lowest concentration at which no growth was observed.

### 3.6. In Vivo Toxicity (Danio rerio FET Test)

Zebrafish (*Danio rerio*) of the AB strain were bred and maintained at the Animal Facility of the Poznan University of Medical Sciences. Adult fish were housed under standard laboratory conditions at 28.5 °C with a 14:10 h light–dark cycle in a recirculating aquaculture system. Following successful spawning, embryos were obtained through natural mating in separate tanks and staged according to established morphological criteria. All procedures and housing conditions adhered to current animal welfare regulations. Adult zebrafish and embryos were kept in E3 medium; non-viable or poor-quality embryos were removed prior to experimentation [37,38].

Under current legislation, studies involving *Danio rerio* larvae up to 120 hpf (hours post fertilization) do not require approval from an animal ethics committee. The experiment was carried out in accordance with published methodologies and based on the principles of OECD Test Guideline No. 236, with modifications that included omitting daily medium exchange and using 10 embryos per well in a 6-well plate [39].

Freshly collected embryos were distributed into 6-well plates at a density of 10 embryos per well, with 3 wells per experimental group. Embryos were maintained in E3 medium and exposed to nanoformulations A-E, free compounds 20-hydroxyecdysone and resveratrol, and dichloroaniline serving as a positive control. The tested concentrations ranged from 0 to 20 µM. Observations were made at 24, 48, 72, and 96 hpf. Mortality and sub-lethal effects were recorded, including lack of embryo development, lack of heart rhythm, lysis, lack of hatching, yolk sac swelling, pericardial edema, morphological deformities, and lack of spontaneous movements.

### 3.7. ^1^H Nuclear Magnetic Resonance

The spin–lattice relaxation time T_1_ and the spin–spin relaxation time T_2_ were recorded.

Solid-state ^1^H NMR is a sensitive technique that enables molecular-level investigation of molecular dynamics through analysis of the spin–lattice relaxation time T_1_ and the spin–spin relaxation time T_2_ of the proton in the laboratory frame. The spin–lattice relaxation time T_1_ characterizes the efficiency of energy transfer from the nuclear spin system to surrounding molecules. In contrast, the spin–spin relaxation time T_2_ relates to energy-conserving interactions between nuclei, leading to the gradual dephasing of spinning dipoles and the exponential decay of magnetization within the transverse (xy) plane.

The efficiency of transverse relaxation strongly depends on molecular mobility. Increased molecular motion leads to more efficient motional averaging of dipole–dipole interactions, resulting in longer T_2_ relaxation times and slower decay of transverse magnetization. In contrast, restricted molecular mobility enhances local dipolar interactions, causing faster dephasing processes and consequently shorter T_2_ relaxation times. Therefore, T_2_ relaxation behaviour is highly sensitive to differences in molecular organization, rigidity, intermolecular interactions, and dynamic heterogeneity within the investigated systems. T_2_ relaxation behaviour can be determined by fitting the curve to the on-resonance solid echo signal. When multiple dynamic environments are present within the sample, several relaxation components are required to adequately describe the decay behaviour. Typically, the longest T_2_ component corresponds to protons located in more mobile regions, whereas the shortest T_2_ component is associated with more rigid and motionally restricted domains. Intermediate relaxation components, when present, reflect regions with intermediate molecular mobility. Consequently, analysis of T_2_ relaxation provides valuable information regarding molecular dynamics, phase morphology, and the coexistence of rigid and mobile domains within complex supramolecular systems such as transferosomal formulations [31,32].

To investigate molecular dynamics within the investigated transferosomal formulations, proton nuclear magnetic resonance (^1^H NMR) relaxation measurements were performed by determining the spin–lattice relaxation time T_1_ and the spin–spin relaxation time T_2_ in the laboratory frame [40]. The spin–lattice relaxation time T_1_ describes the efficiency of energy transfer between the excited nuclear spin system and the surrounding molecular environment. In contrast, the spin–spin relaxation time T_2_ reflects the loss of phase coherence of transverse magnetization caused by spin–spin interactions between neighbouring nuclei.

^1^H NMR experiments were performed using a pulse spectrometer operating at 30.2 MHz. The proton spin–lattice relaxation time T_1_ in the laboratory frame was determined using a standard saturation recovery pulse sequence n×$\left(n\times 90_{x}^{0}-t-90_{x}^{0}\right)$. Experimental data were fitted using the following equation:

(1) $$M\left(t\right)=M_{0}\left(1-exp\left(-\frac{t}{T_{1}}\right)\right)$$

where M_0_ represents the equilibrium magnetization. All measurements were performed with an estimated uncertainty of a few percent [41]. The experimental setup was based on a high-homogeneity B_1_ 30.2 MHz NMR probe previously developed and validated for relaxation time measurements, providing the field homogeneity and measurement stability required for accurate determination of proton relaxation parameters in solid-state and biological samples.

The spin–spin relaxation time T_2_ in the laboratory frame was measured using a standard spin–echo pulse sequence $(90_{x}^{0}-t-180_{x}^{0}).$ For all experiments, the decay curves were fitted using a combination of Gaussian and Lorentzian functions according to the following equation:

(2) $$M\left(t\right)=M_{0G}exp\left(-{\left(\frac{t}{T_{2G}}\right)}^{2}\right)+M_{0L}exp\left(-\frac{t}{T_{2L}}\right)$$

The Gaussian component corresponds to more rigid and motionally restricted regions of the investigated transferosomal formulations, whereas the Lorentzian component represents more mobile domains characterized by higher molecular mobility. Application of this model enabled characterization of the coexistence of rigid and mobile fractions within the investigated systems and evaluation of the influence of formulation composition on molecular organization and dynamic behaviour.

## 4. Conclusions

At the initial time point (D0), the water-hydrated transferosomes exhibited more uniform particle size values when compared to those hydrated with PBS. All formulations showed appropriate polydispersity indices (PDIs) below 0.3, with most samples displaying PDIs around 0.2. The zeta potential ranged from −47 mV to −25 mV for water-based samples and from −41 mV to −32 mV for PBS-hydrated formulations. The apparent encapsulation efficiency of the active compounds (20-hydroxyecdysone and resveratrol) was approximately 90%, whereas the actual encapsulation efficiency determined after size exclusion chromatography (SEC) was in the range of 25–30%. The stability studies showed that the formulations undergo structural changes that mostly seem to reach a plateau over time. Also, the composition of the formulation has a greater effect than the hydration medium’s pH on the physicochemical parameters over time. The content of the active ingredients decreases over time, but to a much lesser extent than would be expected based on literature findings of the non-encapsulated compounds.

Time-domain NMR relaxation studies revealed that formulation composition significantly influences the local molecular dynamics and membrane fluidity of the transferosomes. Specifically, formulation **C** exhibited the highest molecular mobility, suggesting a more dynamic molecular environment within the vesicular membrane, whereas formulation **D** showed more restricted molecular motion and the highest structural heterogeneity.

Importantly, biological studies revealed the safety and suitability of the designed formulations for potential use in psoriasis; neither of the formulations were cytotoxic in vitro, no significant toxic effects were exerted in the zebrafish larvae model, and the formulations had no effect on the commensal microorganisms of human skin.

While the present work provides extensive information on the physicochemical properties, stability, and safety profile of the developed transferosomes, it does not address drug release behaviour, skin permeation and retention, or efficacy in psoriasis-related models. Evaluation of these parameters would be highly valuable for further formulation development and for establishing the practical therapeutic relevance of the system. Accordingly, these studies are planned as the next stage of research.

Overall, the favourable physicochemical characteristics, stability profile, and preliminary biological safety of the developed transferosomes support further investigation as delivery platforms for psoriasis-related applications.

## Figures and Tables

**Table 1:** Physicochemical parameters of formulations **A**–**D** (particle size, PDI, zeta potential, NTA size, encapsulation efficiency, and pH) measured during 30 days of storage in water and PBS (mean ± SD).

							**20-Hydroxyecdysone**					**Resveratrol**					
**D0**	**Z-Average [nm]**	**SD**	**Polydispersity Index (PDI)**	**SD**	**Zeta Potential [mV]**	**SD**	**EE% SEC**	**SD**	**EE%**	**SD**	**pH**	**EE% SEC**	**SD**	**EE%**	**SD**	**NTA [nm]**	**SD**
A H_2_O D0	100.6	1.5	0.2234	0.0130	−37.93	1.54	-	-	-	-	2.82	-	-	-	-	102.9	32.3
B H_2_O D0	92.8	3.0	0.1729	0.0115	−25.88	1.76	27.3	0.0	98.2	0.0	3.34	-	-	-	-	105.4	28.3
C H_2_O D0	90.7	1.0	0.2058	0.0107	−43.24	0.09	27.6	0.0	99.7	0.0	3.28	-	-	-	-	107.6	27.1
D H_2_O D0	107.7	2.4	0.2498	0.0329	−46.96	2.75	29.0	0.0	98.9	0.0	2.96	50.2	1.8	100.0	2.5	114.6	29.1
A PBS D0	156.7	1.0	0.2619	0.0012	−32.60	4.19	-	-	-	-	7.03	-	-	-	-	135.5	43.6
B PBS D0	87.7	1.3	0.1433	0.0145	−33.13	0.40	28.8	0.0	98.2	0.0	6.95	-	-	-	-	108.0	32.5
C PBS D0	113.8	1.1	0.2431	0.0364	−35.52	1.15	25.5	0.0	99.5	0.0	6.96	-	-	-	-	124.6	39.7
D PBS D0	89.84	0.0	0.2101	0.0258	−41.10	2.95	24.3	0.0	98.5	0.0	6.95	21.8	3.1	100.0	1.4	105.2	30.5
**D7**	**Z-Average [nm]**	**SD**	**Polydispersity Index (PDI)**	**SD**	**Zeta Potential [mV]**	**SD**	**EE% SEC**	**SD**	**EE%**	**SD**	**pH**	**EE% SEC**	**SD**	**EE%**	**SD**	**NTA [nm]**	**SD**
A H_2_O D7	96.6	1.0	0.2016	0.0149	−28.63	0.27	-	-	-	-	2.75	-	-	-	-	152.2	46.7
B H_2_O D7	110	2.2	0.2505	0.0212	−35.97	0.70	26.8	0.0	99.4	0.0	3.19	-	-	-	-	120.6	38.2
C H_2_O D7	95.08	0.3	0.2045	0.0187	−22.85	1.48	21.6	0.0	96.2	0.0	3.24	-	-	-	-	139.4	43.6
D H_2_O D7	131.3	1.7	0.2295	0.0105	−30.47	0.11	27.8	0.0	100.2	0.0	2.96	47.4	1.2	93.4	1.1	142.4	54.5
A PBS D7	153	1.6	0.2978	0.0341	−27.92	0.96	-	-	-	-	6.98	-	-	-	-	194.8	78.8
B PBS D7	96.97	1.5	0.2338	0.0194	−23.82	0.74	24.1	0.0	99.8	0.0	7.02	-	-	-	-	159.7	62.9
C PBS D7	119.6	1.1	0.3214	0.0330	−29.04	0.18	25.4	0.0	99.4	0.0	7.05	-	-	-	-	150.0	53.3
D PBS D7	91.44	1.7	0.2200	0.0145	−7.029	1.02	20.1	0.0	98.1	0.0	7.00	21.0	0.9	87.5	1.8	153.0	59.7
**D14**	**Z-Average [nm]**	**SD**	**Polydispersity Index (PDI)**	**SD**	**Zeta Potential [mV]**	**SD**	**EE% SEC**	**SD**	**EE%**	**SD**	**pH**	**EE% SEC**	**SD**	**EE%**	**SD**	**NTA**	**SD**
A H_2_O D14	109.4	6.0	0.2483	0.0214	−20.38	4.28	-	-	-	-	2.84	-	-	-	-	138.8	61.0
B H_2_O D14	95.31	0.2	0.1606	0.0018	−27.59	1.30	20.0	0.0	100.3	0.0	3.35	-	-	-	-	107.3	28.3
C H_2_O D14	117.3	8.9	0.2755	0.0444	−32.99	6.17	21.5	0.0	101.8	0.0	3.31	-	-	-	-	116.8	34.8
D H_2_O D14	116.7	1.1	0.2169	0.0113	−28.91	1.52	21.9	0.0	101.3	0.0	3.03	41.1	1.0	88.9	2.3	113.3	38.2
A PBS D14	154.9	1.7	0.2606	0.0087	−33.49	3.96	-	-	-	-	6.95	-	-	-	-	139.8	47.0
B PBS D14	96.6	3.3	0.2198	0.0250	−6.90	1.00	20.8	0.0	95.1	0.0	6.97	-	-	-	-	107.8	30.9
C PBS D14	156.3	5.7	0.3195	0.0448	−27.06	1.52	20.7	0.0	99.0	0.0	7.01	-	-	-	-	130.4	48.0
D PBS D14	111.7	1.5	0.3271	0.0403	−17.16	1.77	16.7	0.0	99.0	0.0	6.96	20.6	1.5	88.5	3.3	96.5	32.2
**D21**	**Z-Average [nm]**	**SD**	**Polydispersity Index (PDI)**	**SD**	**Zeta Potential [mV]**	**SD**	**EE% SEC**	**SD**	**EE%**	**SD**	**pH**	**EE% SEC**	**SD**	**EE%**	**SD**	**NTA**	**SD**
A H_2_O D21	118.6	11.8	0.2405	0.0299	−13.73	0.52	-	-	-	-	3.21	-	-	-	-	98.1	38.5
B H_2_O D21	101.5	7.2	0.1948	0.0324	−34.01	0.40	19.2	0.0	90.4	0.0	3.59	-	-	-	-	86.2	31.5
C H_2_O D21	101.8	1.5	0.2414	0.0173	−19.02	1.33	21.3	0.0	99.7	0.0	3.58	-	-	-	-	96.1	32.1
D H_2_O D21	139.6	6.7	0.2211	0.0423	−14.64	0.58	20.0	0.0	99.0	0.0	3.33	33.6	0.4	88.0	4.7	103.1	36.9
A PBS D21	186.2	5.7	0.2431	0.0668	−16.10	0.53	-	-	-	-	7.10	-	-	-	-	143.8	57.2
B PBS D21	97.72	1.2	0.1961	0.0128	−33.95	1.04	16.8	0.0	92.4	0.0	7.11	-	-	-	-	115.0	47.2
C PBS D21	180	4.4	0.2904	0.0509	−28.65	0.25	20.2	0.0	94.2	0.0	7.12	-	-	-	-	124.5	44.6
D PBS D21	113.7	15.3	0.2534	0.0463	−14.21	0.68	16.2	0.0	97.7	0.0	7.10	19.9	0.5	89.3	1.5	91.2	30.9
**D30**	**Z-Average [nm]**	**SD**	**Polydispersity Index (PDI)**	**SD**	**Zeta Potential [mV]**	**SD**	**EE% SEC**	**SD**	**EE%**	**SD**	**pH**	**EE% SEC**	**SD**	**EE%**	**SD**	**NTA**	**SD**
A H_2_O D30	99.47	0.5	0.2032	0.0267	−21.59	6.67	-	-	-	-	3.11	-	-	-	-	112.5	40.5
B H_2_O D30	160.6	2.1	0.2060	0.0148	−39.18	0.78	16.1	0.0	101.4	0.0	3.54	-	-	-	-	146.2	48.4
C H_2_O D30	135	1.8	0.2820	0.0131	−34.38	2.67	18.3	0.0	100.7	0.0	3.33	-	-	-	-	115.4	31.1
D H_2_O D30	129.2	7.9	0.2537	0.0103	−27.27	2.21	19.5	0.0	97.4	0.0	3.27	8.7	0.2	85.4	2.1	131.6	35.1
A PBS D30	152.5	1.3	0.2570	0.0075	−34.15	2.27	-	-	-	-	7.11	-	-	-	-	143.9	51.1
B PBS D30	94.38	0.6	0.2057	0.0159	−38.37	14.36	16.6	0.0	91.8	0.0	7.15	-	-	-	-	110.7	38.0
C PBS D30	157.5	4.4	0.3037	0.0665	−46.70	7.96	18.9	0.0	93.5	0.0	7.14	-	-	-	-	139.9	55.7
D PBS D30	91.9	1.0	0.2210	0.0056	−26.19	7.41	14.8	0.0	97.8	0.0	7.12	13.7	0.7	85.7	2.2	97.9	31.8

**Table 2:** Antimicrobial activity of formulations **A**–**D** expressed as minimum inhibitory concentration (MIC, mM) and minimum bactericidal concentration (MBC, mM) against standard and clinical strains of *Staphylococcus epidermidis* and *Staphylococcus aureus*.

Bacterial Strain	*S. epidermidis* (Standard)		*S. epidermidis* (Clinical)		*S. aureus* (Standard)		*S. aureus* (Clinical)	
[mM]	MIC	MBC	MIC	MBC	MIC	MBC	MIC	MBC
Formulation **A**	>0.5	>0.5	>0.5	>0.5	>0.5	>0.5	>0.5	>0.5
Formulation **B**	>0.5	>0.5	>0.5	>0.5	>0.5	>0.5	>0.5	>0.5
Formulation **C**	>0.5	>0.5	>0.5	>0.5	>0.5	>0.5	>0.5	>0.5
Formulation **D**	>0.5	>0.5	>0.5	>0.5	>0.5	>0.5	>0.5	>0.5

**Table 3:** Representative photographs of zebrafish larvae (*Danio rerio*) at 96 hpf exposed to the tested samples. Images were acquired using a Leica MZ10F microscope (Leica Mircosystems, Heerbrugg, Switzerland) at 3× magnification. Corr.—corresponding to the given concentration (only formulation, no APIs). Numbers presented in the table represent the percentage of live larvae per total larvae. Reported abnormalities include def—body deformities, yse—yolk sac edema, and pe—pericardial edema.

Sample	Representative Photo (96 hpf)	Live Larvae per Total Larvae (96 hpf)
E3 buffer (no compound, no nanoformulation)	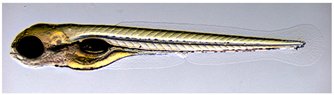	93% (28/30)
Dichloroaniline (positive control), conc. 25 µM (4 mg/L)	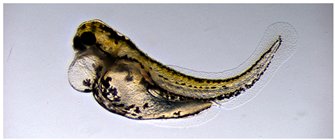	80% (24/30) 13 yse 15 pe 11 def
Resveratrol (API), 20 µM	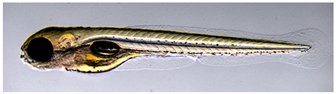	100% (30/30)
20-Hydroxyecdysone (API), 20 µM	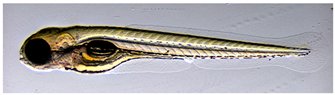	97% (29/30)
A Corr. 20 µM	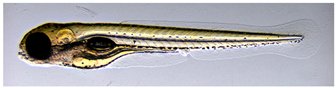	97% (29/30)
B (20-HE, 20 µM)	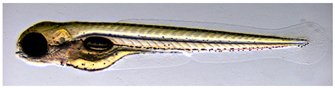	100% (30/30) 1 yse 1 pe 1 def
C (20-HE, 20 µM)	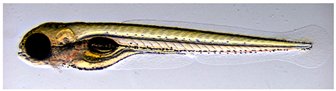	97% (29/30)
D (20-HE, res, 20 µM)	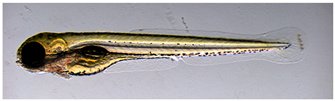	100% (30/30)

**Table 4:** Spin–lattice relaxation time T_1_ and spin–spin relaxation parameters T_2_ determined in the laboratory frame for the investigated transferosomal formulations.

Material	T_1_ (s)	$M_{0G}\;(\%)$	T_2G_ (ms)	$M_{0L}\;(\%)$	T_2L_ (ms)
Formulation **A**	2.30	79	198	21	434
Formulation **B**	2.21	85	214	15	519
Formulation **C**	2.17	88	227	12	765
Formulation **D**	2.30	70	227	30	199

**Table 5:** Detailed composition of transferosomal formulations **A**–**D**, expressed as molar concentrations (mM) and mass concentrations (mg/mL).

	Phospholipon 80H	Tween 20	20-HE	Resveratrol
Formulation **A**	20 mM 15.2 mg/mL	1 mM 1.23 mg/mL	-	-
Formulation **B**	20 mM 15.2 mg/mL	1 mM 1.23 mg/mL	1 mM 0.48 mg/mL	-
Formulation **C**	20 mM 15.2 mg/mL	3 mM 3.68 mg/mL	1 mM 0.48 mg/mL	-
Formulation **D**	20 mM 15.2 mg/mL	1 mM 1.23 mg/mL	1 mM 0.48 mg/mL	1 mM 0.228 mg/mL

**Table 6:** A summary of validation parameters for the HPLC method used for EE determination.

Parameters	Acceptance Criteria	Results	
		Resveratrol	20-Hydroxyecdysone
Selectivity Influence of interfering substances	Separation of the active substance peak from the peaks of formulation ingredients	t_R_ = 6.0 min	t_R_ = 4.7 min
		Lack of interference peaks at 334 nm	Acceptable blank peaks at 245 nm: t_R_ 6.3–6.9, 8.7 and 10.2 min
Linearity Correlation coefficient r for the equation y = ax	r ≥ 0.990	Acceptable y = (42,999.5 ± 505.7)x; r = 0.9998	Acceptable y = (14,499.2 ± 434.2)x; r = 0.9979
Repeatability - intra-day - inter-day	RSD ≤ 5%	Acceptable RSD = 0.93% RSD = 0.87%	Acceptable RSD = 0.06% RSD = 0.09%
Calibration range, mg/mL		0.0025–0.05	0.0013–0.008
Limits of detection and quantitation		DL = 0.0008 QL = 0.0025	DL = 0.0004 QL = 0.0013

## Data Availability

The data are contained within the article.
